# Screening and understanding adolescents with money worries via a web‐based stress check: A latent class analysis

**DOI:** 10.1002/pcn5.70274

**Published:** 2025-12-26

**Authors:** Yurika Namihira, Miyuki Furukawa, Ayako Tsuchiya, Yoshikazu Noda, Seiichiro Hori, Takako Koshiba, Hironori Shimada, Eiji Shimizu

**Affiliations:** ^1^ United Graduate School of Child Development The University of Osaka, Kanazawa University, Hamamatsu University School of Medicine, Chiba University and University of Fukui Osaka Japan; ^2^ Research Center for Child Mental Development Chiba University Chiba Japan; ^3^ Faculty of Nursing Josai International University Chiba Japan; ^4^ Department of Nursing, Faculty of Human Care at Makuhari Tohto University Chiba Japan; ^5^ Faculty of Human Sciences Waseda University Saitama Japan; ^6^ Department of Cognitive Behavioral Physiology, Graduate School of Medicine Chiba University Chiba Japan

**Keywords:** adolescent psychiatry, early intervention, mental health, money worries, school‐based SDOH screening

## Abstract

**Aim:**

This study investigates patterns of stress responses (SR), stressors, and social support (SS) among high school students using a dataset from a web‐based stress check system, aiming to identify students experiencing high stress or money worries.

**Methods:**

After obtaining informed consent from both students and their parents, high school students were assessed using the Public Health Research Foundation‐Type Stress Inventory and additional questions to screen for money worries. Latent class analysis was used to identify stress patterns, and multinomial logistic regression was applied to explore risk factors.

**Results:**

Among all the students (*n* = 6894), five latent classes were identified: “high SR & low SS” (17.8%), “high SR & SS” (14.2%), “moderate SR” (29.8%), “low SR & SS” (11.8%), and “minimal SR” (26.4%). Students with money worries (*n* = 630) were 3.78 times more likely to be in the “high SR & low SS” class compared to the “minimal SR” class (odds ratio [OR] = 3.78, *p* < 0.001). Further analysis revealed that students with money worries could be divided into three groups: “low SR” (40.5%), “moderate SR” (28.4%), and “high SR” (31.1%). The low SR group had high SS, while the moderate and high SR groups reported lower SS.

**Conclusion:**

Our findings suggest that money worries may be linked to higher stress levels among students. A web‐based child stress check could be a valuable tool for identifying students at risk of mental health issues and money worries. Further research is necessary to explore stress‐check systems for adolescents.

## INTRODUCTION

Children today face more mental health crises and financial hardships than previous generations, largely due to global uncertainty and the impact of the Coronavirus disease 2019 (COVID‐19) pandemic.^1^ In Japan, although the relative child poverty rate improved from 14.0% in 2018 to 11.5% in 2021,^2^, ^3^, ^4^ the average household income has declined since 2022, and the number of child suicides and cases of chronic absenteeism has reached the highest levels on record.^5^, ^6^ Experiencing poverty during childhood and adolescence can lead to chronic stress, negatively affecting a child's long‐term well‐being on a broad scale.^7^ This not only harms physical and mental health in adulthood^8^ but also contributes to the intergenerational transmission of poverty.^9^, ^10^ For high school students, poverty can serve as a significant source of stress, impacting mental health. It is linked to poor academic performance, lack of access to leisure activities,^11^, ^12^ and psychological burdens, such as anxiety, shame, and fear of social exclusion in friendships.^13^ Moreover, it can lead to experiences of social exclusion, such as difficulty maintaining friendships^14^ and suspension or expulsion from school.^15^, ^16^ These factors increase the risk of lower income in young adulthood^17^ and unintended pregnancies.^18^

However, children can develop skills and abilities even in harsh environments, potentially utilizing these skills to their advantage.^19^ Some studies have shown that certain children can maintain a healthy allostatic load, demonstrate mental resilience, and achieve strong academic results.^20^, ^21^, ^22^ In recent years, an increasing number of studies have focused on protective factors.^23^ For example, social support at various levels—such as support from family,^24^ friends,^25^ positive relationships with teachers,^26^ school climate,^27^ and the local community^28^—is crucial in mitigating the negative impact of stressors in poverty‐related environments, even though they can also act as stressors.^29^

In research on stress management, cognitive behavioral therapy‐based (CBT‐based) interventions are commonly used and are effective in reducing depression or anxiety in adolescents at risk,^30^, ^31^ including those affected by poverty.^32^, ^33^ For adolescents living in poverty, stress management interventions are especially important in schools due to limited access to medical care.^34^ Although free medical care for children is widespread in Japan, a high percentage of children from poor families cannot consult a doctor due to their parents' long working hours.^35^ Efforts to enhance health and educational equity have been made through various means, such as trauma‐informed care, school‐wide support, subsidies for uniforms and extracurricular activities, and free school lunches. Additionally, data‐driven educational initiatives using health data have been implemented.^36^ However, these efforts have largely focused on primary and secondary education, with limited attention given to financial stress in high schools. While some intervention studies have highlighted the impact of poverty on dropout prevention in high schools,^37^ few comprehensive studies have incorporated mental health indicators, and reports on specific support methods remain necessary.

This study addresses two objectives by adding simple questions to regular online web‐based stress checks: (1) to identify students with high‐stress levels and financial concerns, and (2) to analyze the characteristics of potential classes that should be prioritized for support. For objective (2), we will use latent class analysis (LCA) to examine the stressors likely to be experienced in school and the perceived social support. Our main hypothesis is that financial worries are associated with high‐stress levels in adolescents.

LCA is a person‐centered approach that estimates latent subclasses based on related characteristics derived from a few questions. It is an effective method for obtaining useful results without increasing participant burden through regular stress checks. Multinomial logistic regression was used to examine the factors associated with each latent class identified through LCA.

## METHODS

### Web‐based child stress check study

This study is part of the larger “Web‐based Child Stress Check Study,” which involves a survey administered by Chiba University's Research Center for Child Mental Development in collaboration with local education committees. The project, ongoing since 2021, aims to reduce child suicides. The study protocol was approved by the Ethics Committee of the Chiba University Graduate School of Medicine (ID: M10631). Informed consent was obtained from parents before the study commenced for children aged under 18 years. Children, including those aged over 18 years, participate using their own digital devices after reviewing information about the study and providing informed consent. Based on their responses to a questionnaire, the system immediately provides respondents' stress levels and related advice based on CBT to both students and school teachers. The results are displayed on a feedback screen, which is viewable by students and teachers. The feedback screens are printable and include the phone number for the national child SOS consultation service, as well as QR codes linking to short videos of simplified self‐CBTs, such as the Japanese versions of the Three Good Things Exercise “Pojiren”^38^ and the 5‐min CBT exercise “Kokoren”.^39^ Teachers and other education and health professionals can use the feedback sheet to provide individualized educational consultations.

### Participants

Data for the present study were collected from August to December 2022 and involved 7032 high school students (ages 16–18 years) attending public schools in a single municipality. This was a cross‐sectional study. We excluded 60 students (0.85%) due to duplicated answers, eight students (0.11%) who had missing grade data, and 47 students (0.67%) who had missing gender data. Additionally, 23 students (0.33%) who provided long‐string responses to all items were excluded for careless responses to maintain the validity of the online experiment.^40^, ^41^ After excluding a total of 138 students, 6894 students' responses (98.0%) were included in the analysis.

### Measures

#### The public health research foundation‐type stress inventory (PSI)

The PSI is a comprehensive assessment tool that evaluates children's daily mental health, developed by Sakano and colleagues.^42^ As part of a preventative approach for the early detection and intervention of children at high risk of issues such as chronic absenteeism and bullying, it is an objective and user‐friendly inventory for assessing children's mental health. The PSI consists of three components to measure children's stress responses, stressors, and perceived social support: (A) stress response subscale (SR), which measures mental health conditions, including depression, anxiety, moodiness, anger, and helplessness; (B) stressor subscale (ST), which examines stress sources such as teacher relationships, friendships, academic performance and challenges, and future career paths; (C) social support subscale (SS), which assesses perceived social support, including help from family, homeroom teachers, and friends. In this subscale, a higher score suggests a higher social support level. The scale uses a four‐item Likert scale, ranging from 0 to 3 (Table 1). The version of the PSI designed for high school students was adopted for this study. Cronbach's alpha values for this study were 0.94, 0.80, and 0.93 for the SR, ST, and SS subscales.

**Table 1:** Public health research foundation‐type stress inventory (PSI) high school student questionnaire.

Subscales	Items	
Stress response subscale (SR)	(A) About your recent feelings and physical condition. (Options: does not apply at all = 0, does not apply very well = 1, applies a little = 2, applies well = 3)	
	Item #	Subscale category #
	1. I feel sad.	DEP 1
	2. I tend to become angry.	IRR 1
	3. I lack confidence in many things.	HEL 1
	4. I'm somewhat worried.	DEP 2
	5. I am unable to control emotions.	IRR 2
	6. I lack perseverance.	HEL 2
	7. I feel down.	DEP 3
	8. I am displeased with things.	IRR 3
	9. I am thinking about things that are not good.	HEL 3
	10. I feel like crying.	DEP 4
	11. I am angry.	IRR 4
	12. I cannot concentrate on anything.	HEL 4
	13. I want to be consoled.	DEP 5
	14. I am irritable.	IRR 5
	15. I can't get my story or actions straight.	HEL 5
Stressor subscale (ST)	(B) How often in the last 2 months have you experienced any of the following? (Options: not at all = 0, not often = 1, sometimes = 2, often = 3)	
	Item #	Subscale category #
	1. The teacher scolded me even though what happened was not my fault.	SCH 1
	2. My friend was violent.	FRI 1
	3. I did not understand the content of a class.	ACA 1
	4. I was asked to change my career choice.	CAR 1
	5. Teachers compared me negatively with other students.	SCH 2
	6. My friends said bad things about me.	FRI 2
	7. I could not answer when I was called upon in class.	ACA 2
	8. I was concerned with respect to my career path.	CAR 2
	9. The teacher imposed his or her ideas on me.	SCH 3
	10. A friend asked me to run an errand.	FRI 3
	11. I received worse grades than my friends.	ACA 3
	12. I talked about career paths with friends.	CAR 3
	13. The teacher did not respond to me in a friendly manner.	SCH 4
	14. My friends suspected me of doing something even though it was not my fault.	FRI 4
	15. I did not get the grades I expected.	ACA 4
	16. I talked about career paths with my family (e.g., my parents).	CAR 4
Social support subscale (SS)	(C) How much do you feel that the people around you are usually helpful to you? (Options: I think not = 0, I think probably not = 1, I think probably so = 2, I think surely so = 3)	
	Item #	Subscale category #
	1. They notice right away if you are not feeling well and encourage you. Your family member.	SHOM 1
	2. They notice right away if you are not feeling well and encourage you. Your teachers.	SSCH 1
	3. They notice right away if you are not feeling well and cheer you up. Your friends.	SFRI 1
	4. If you make any mistakes, they gently help you out. Your family member.	SHOM 2
	5. If you make any mistakes, they gently help you out. Your teachers.	SSCH 2
	6. If you make any mistakes, they gently help you out. Your friends.	SFRI 2
	7. They usually know how you feel. Your family member.	SHOM 3
	8. They usually know how you feel. Your teachers.	SSCH 3
	9. They usually know how you feel. Your friends.	SFRI 3
	10. If they know you are worried, they will tell you what to do. Your family member.	SHOM 4
	11. If they know you are worried, they will tell you what to do. Your teacher.	SSCH 4
	12. If they know you are worried, they will tell you what to do. Your friends.	SFRI 4

Abbreviations: ACA, academic; CAR, career path; DEP, depression/anxiety; FRI, friends; HEL, helplessness; IRR, irritability; SCH, school teacher; SFRI, support from friends; SHOM, support at home; SSCH, support from school teacher.

The PSI consists of three original scales: the psychological stress response scale (SRS‐18) developed by Suzuki et al.,^43^ the School Stressor Scale for high school students developed by Shimada et al.,^44^ and the Social Support Scale for junior high school students developed by Okayasu et al.^45^ These scales were created by reducing the number of items from the original versions as much as possible while maintaining the reliability of the original scales. The goal was to ensure that the items could be answered by respondents quickly and easily and that they could be easily tabulated by users, such as teachers.

Combining these three scales in the PSI enables a deeper understanding of individual students' circumstances in educational and clinical support, viewed through the lens of the stress‐buffering effect of social support by Cohen and Wills^46^ and the cognitive appraisal of stress responses within the stress theory framework by Lazarus and Folkman.^47^

#### Screening for high stress level

We established the criteria for high stress and cutoff scores based on stress‐check programs in Japanese workplaces^48^ and the cutoff values for the brief job stress questionnaire (BJSQ), which the Ministry of Health, Labour and Welfare recommends.^49^ In the BJSQ, Criterion A is set at 66.4% or higher of the total score on the SR subscale (77 points or higher out of 116 points), Criterion B is set at 54.3% or higher of the total score on the SR subscale (63 points or higher out of 116 points), and 73.1% or higher of the total score on the stressor and SS subscales (76 points or higher out of 104 points).

Based on these criteria of the BJSQ, we defined high stress in the PSI as follows: In the PSI, Criterion A was set at 66.7% or higher of the total score on the SR subscale (30 points or higher out of 45 points), whereas Criterion B was set at 53.3% or higher of the total score on the SR subscale (24 points or higher out of 45 points) and 72.6% or higher of the total score on the stressor and SS subscales (61 points or higher out of 84 points). For the Criterion B setting, we use the lowness of social support—the full score minus the social support score—to identify students with the lowest level of social support.

#### Additional question: Money worries

In addition to the PSI, we included a multiple‐choice question on worries. The question, “Are there any issues that are troubling you?” offered seven response options: “Family illness or disability,” “Having a family member to take care of,” “Livelihood or money,” “Having little free time for myself,” “School grades and career paths,” “Other,” and “No worries.” Students who selected “Livelihood or money” were identified as having money worries, which may indicate relative poverty.

### Statistical analysis

To address the first study objective, LCA was used to examine the participants overall, exploring latent classes based on the PSI scores. LCA is a person‐centered approach that identifies hidden heterogeneity within a sample, providing a deeper understanding of individuals within a larger class.^50^ For model estimation, variables were dichotomized based on the presence or absence of each item. Robust maximum likelihood estimation was used, with 1200 sets of random start values. The number of latent classes was determined using the following statistical criteria: (1) Akaike's information criterion (AIC), Bayesian information criterion (BIC), and sample‐size‐adjusted BIC (SABIC), with smaller values indicating better model fit; (2) the Lo–Mendell–Rubin likelihood ratio test (LMR‐LRT) and the bootstrapped likelihood ratio test (BLRT), where significant values indicate that a model with K classes is better than one with K‐1 classes; and (3) entropy, where values exceeding 0.8 indicate high classification accuracy (often greater than 90%).^51^ We used 100 random starts with 20 bootstrap draws for each comparison to ensure reliable estimates. Furthermore, theoretical interpretation requires that identified classes be explained theoretically and their implications for practice be considered.^52^ Second, the $\chi^{2}$ test and analysis of variance (ANOVA) were conducted to compare categorical variables and PSI subscale scores between groups using Stata/BE 18.0 (Stata Corp LP, College Station, TX, USA).

In addition, to compare PSI subscale scores of students with money worries and without any worries, the effect sizes were calculated using Cramer's V for categorical variables.^53^ Cramér's V values were interpreted as follows: 0–0.1, weak association; >0.1–0.3, moderate association; >0.3–0.5, strong association; and >0.5, very strong association. Eta‐squared (*η* ^2^) was used for PSI scores, and the effect size values can be interpreted as follows: 0–0.06, small; 0.06–0.14, medium; and >0.14, large.

Finally, multinomial regression analysis using a bias‐adjusted three‐step approach^54^ was performed, where latent classes served as the dependent variable to explore differences across classes by grade, gender, and money worries. Statistical tests adopted a significance level of *α* = 0.05. LCA and multinomial logistic regression analyses were conducted using Mplus 8.10 (Muthén & Muthén).

To address the second study objective, LCA and multinomial logistic regression were used in the same manner as for the sample with money worries, particularly to explore their specific circumstances regarding stressors and perceived social support. Then, in the money worries class, the degree of separation for each PSI item in the LCA results was examined.^55^, ^56^

## RESULTS

A total of 6894 students' responses were analyzed (Table 2). Of these, 3080 (44.7%) were male, 3540 (51.3%) were female, and 274 (4.0%) identified as another gender. Of all the students, 874 (12.7%) were classified as high‐stress Class A, and 2 (0.03%) were classified as high‐stress Class B. The students with money worries (*n* = 630) are detailed in Table 3. Our results show that approximately 1 in 11 students reported having money worries. Among students with money worries, 126 (20.0%) were classified as high‐stress Class A, and none (0%) were classified as high‐stress Class B.

**Table 2:** Sociodemographic information of participants and class comparison for the PSI (*n* = 6894).

Demographic variables	Overall	Class 1	Class 2	Class 3	Class 4	Class 5	Statistics	*p*
	*n* = 6894 *n* (%)	High SR & low SS 1227 (17.8%)	High SR & SS 977 (14.2%)	Moderate SR 2053 (29.8%)	Low SR & SS 814 (11.8%)	Minimal SR 1823 (26.4%)	(*χ* ^*2*^/*F*)	
Grade								
Grade 10	2876 (41.7)	517 (42.1)	364 (37.3)	874 (42.6)	351 (43.1)	770 (42.2)	24.159	<0.01^a^
Grade 11	2041 (29.6)	392 (31.9)	302 (30.9)	588 (28.6)	257 (31.6)	502 (27.5)		
Grade 12	1977 (28.7)	318 (25.9)	311 (31.8)	591 (28.8)	206 (25.3)	551 (30.2)		
Gender								
Male	3080 (44.7)	493 (40.2)	316 (32.3)	831 (40.5)	508 (62.4)	932 (51.1)	241.718	<0.001^a^
Female	3540 (51.3)	666 (54.3)	623 (63.8)	1150 (56.0)	266 (32.7)	835 (45.8)		
Other	274 (4.0)	68 (5.5)	38 (3.9)	72 (3.5)	40 (4.9)	56 (3.1)		
Results for stress screening								
Normal stress	6018 (87.3)	754 (61.5)	576 (59.0)	2051 (99.9)	814 (100)	1823 (100)	2100.000	<0.001^a^
High stress (A)	874 (12.7)	471 (38.4)	401 (41.0)	2 (0.1)	0 (0.0)	0 (0.0)		
High stress (B)	2 (0.03)	2 (0.20)	0 (0.00)	0 (0.00)	0 (0.00)	0 (0.00)		
Money worry	630 (9.1)	195 (0.31)	98 (0.16)	168 (0.27)	72 (0.11)	97 (0.15)	102.6825	<0.001^a^
**Mean (SD) of total and subscales**								
Scores of PSI stress response (SR)								
Total	16.55 (10.74)	28.11 (6.64)	29.09 (5.30)	16.13 (5.06)	10.05 (6.64)	5.40 (5.07)	4655.303	<0.001^b^
Depression	5.36 (3.98)	9.27 (3.02)	9.81 (2.59)	5.16 (2.41)	3.05 (2.52)	1.61 (1.84)	3010.206	<0.001^b^
Helplessness	6.66 (4.02)	10.37 (2.65)	10.49 (2.52)	7.25 (2.54)	4.26 (2.96)	2.52 (2.18)	2936.552	<0.001^b^
Irritability	4.52 (3.93)	8.47 (3.34)	8.80 (2.76)	3.72 (2.52)	2.73 (2.70)	1.27 (1.84)	2272.67	<0.001^b^
Scores of PSI stressor (ST)								
Total	12.16 (6.48)	16.98 (6.92)	14.89 (5.69)	13.11 (4.92)	9.41 (5.91)	7.60 (4.66)	677.429	<0.001^b^
Friends	0.93 (1.66)	1.98 (2.34)	1.30 (1.89)	0.75 (1.33)	0.88 (1.48)	0.27 (0.80)	232.043	<0.001^b^
School	1.17 (1.98)	2.64 (2.83)	1.21 (1.88)	0.95 (1.59)	1.26 (1.92)	0.35 (0.92)	256.328	<0.001^b^
Academic	5.15 (3.01)	6.79 (2.77)	6.42 (2.67)	5.85 (2.61)	3.89 (2.80)	3.14 (2.50)	533.608	<0.001^b^
Career	4.91 (2.65)	5.57 (2.61)	5.96 (2.44)	5.56 (2.28)	3.38 (2.46)	3.84 (2.57)	264.185	<0.001^b^
Scores of PSI social support (SS)								
Total	23.88 (8.39)	14.28 (6.07)	25.48 (4.56)	26.58 (4.63)	14.17 (7.24)	30.77 (4.68)	2152.075	<0.001^b^
Friends	8.63 (3.06)	6.05 (3.32)	8.94 (2.16)	9.48 (1.93)	5.76 (3.55)	10.52 (1.75)	724.884	<0.001^b^
School teacher	6.95 (3.35)	3.18 (2.33)	7.98 (1.91)	7.80 (2.26)	3.22 (2.45)	9.64 (1.97)	2218.198	<0.001^b^
Family	8.30 (3.43)	5.05 (3.42)	8.56 (2.82)	9.30 (2.28)	5.19 (3.57)	10.60 (1.71)	1025.032	<0.001^b^

^a^ Chi‐square (*χ* ^2^).

^b^ Analysis of variance (ANOVA).

**Table 3:** Sociodemographic information of participants with financial worry and class comparison for the PSI (*n* = 630).

	Class 1: Moderate SR		Class 2: High SR		Class 3: Low SR		Statistics (*F*/*χ* ^*2*^)	*p*
	179 (28.4%)		196 (31.1%)		255 (40.5%)			
Grade								
Grade 10	60	(33.5)	75	(38.3)	87	(34.1)	3.9675	0.41
Grade 11	53	(29.6)	54	(27.6)	61	(23.9)		
Grade 12	66	(36.9)	67	(34.2)	107	(42.0)		
Gender								
Male	95	(53.1)	71	(36.2)	126	(49.4)	13.5703	<0.01^a^
Female	79	(44.1)	112	(57.1)	118	(46.3)		
Other	5	(2.8)	13	(6.6)	11	(4.3)		
Results for stress screening								
Normal stress	172	(61.5)	77	(0.4)	255	(59.0)	295.7734	<0.001^a^
High stress (A)	7	(38.4)	119	(0.6)	0	(41.0)		
High stress (B)	0	(0.2)	0	(0.0)	0	(0.0)		
**Mean (SD) of total and subscales**								
Scores of PSI stress response (SR)								
Total	18.96	(6.50)	31.07	(5.38)	12.47	(6.91)	538.992	<0.001^b^
Depression	5.98	(3.09)	10.19	(2.63)	3.77	(2.70)	324.871	<0.001^b^
Helplessness	7.54	(2.96)	11.16	(2.43)	5.47	(3.02)	253.729	<0.001^b^
Irritability	5.43	(3.27)	9.71	(2.76)	3.24	(2.85)	299.421	<0.001^b^
Scores of PSI stressor (ST)								
Total	13.04	(6.50)	17.23	(6.37)	11.27	(5.42)	55.409	<0.001^b^
Friends	2.02	(2.34)	2.48	(2.69)	0.85	(1.55)	37.726	<0.001^b^
School	1.44	(1.90)	2.35	(2.40)	0.77	(1.34)	36.714	<0.001^b^
Academic	5.17	(2.96)	6.73	(2.79)	4.64	(2.68)	33.057	<0.001^b^
Career	4.42	(2.45)	5.68	(2.43)	5.01	(2.29)	12.502	<0.001^b^
Scores of PSI social support (SS)								
Total	14.07	(6.06)	18.97	(7.44)	27.74	(4.52)	361.978	<0.001^b^
Friends	6.08	(3.22)	7.26	(3.28)	9.70	(1.84)	115.595	<0.001^b^
School teacher	3.76	(2.62)	5.20	(3.23)	8.27	(2.28)	189.293	<0.001^b^
Family	4.23	(3.01)	6.52	(3.68)	9.77	(1.81)	269.480	<0.001^b^

^a^ Chi‐square (*χ* ^2^).

^b^ Analysis of variance (ANOVA).

### Overall student sample

#### Model selection and labeling of the identified latent class

**FIGURE 1:**
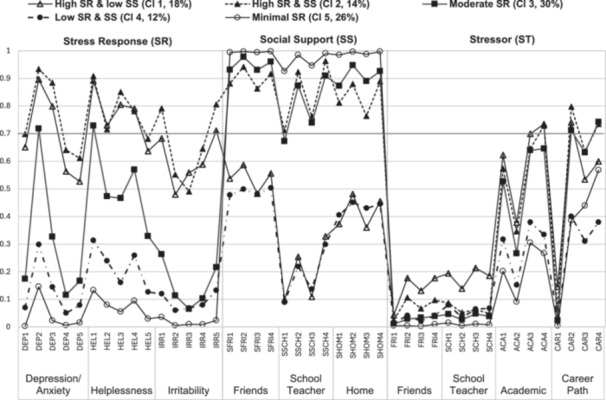
Latent classes of the sample of the overall students (*n* = 6894). ACA, academic; CAR, career path; DEP, depression/anxiety; FRI, friends; HEL, helplessness; IRR, irritability; SCH, school teacher; SFRI, support from friends; SHOM, support at home; SSCH, support from school teacher.

The optimal number of latent classes for the overall student sample of PSI was determined by running a series of LCAs, with the number of latent classes gradually increasing from one to seven. Table 4 presents a summary of the model fit indices for all tested LCAs. In the overall sample, although the AIC, BIC, and SABIC values decreased with an increasing number of latent classes, the LMR‐LRT indicated that a five‐class model fit the data better than a six‐class model (*p* < 0.001). While the entropy of the five‐class model (0.881) was slightly lower than that of the other models, it was preferred over the seven‐class model based on a balance of model fit. Table 2 and Figure 1 show the latent class membership and conditional probability distribution diagrams for each identified class across the 43 items of the PSI. The latent classes were characterized by the conditional item probabilities for the SR and SS subscales. For the overall student sample, class sizes ranged from 11.8% (Class 4, *n* = 814) to 29.8% (Class 3, *n* = 2053) of the total sample (Table 2). As shown in Figure 1, Class 1 and Class 2 exhibited relatively high ratings for items in the SR domain; however, Class 2 also showed high ratings in the SS domain. Class 3 demonstrated moderate ratings for the SR. Class 4 exhibited relatively low ratings in the SR domain, while Class 5 showed minimal variation in the SR domain and high ratings in the SS domain. Based on these patterns, the five classes were labeled as follows: “high SR & low SS” (17.8%), “high SR & SS” (14.2%), “moderate SR” (29.8%), “low SR & SS” (11.8%), and “minimal SR” (26.4%).

**Table 4:** Summary of the latent class analysis fit indices.

Classes	AIC	BIC	SABIC	LMR‐LRT *p* value	BLRT *p* value	Entropy
Overall						
1	305883.345	306177.396	306040.752	‐	‐	‐
2	269981.524	270576.465	270300.000	0.000	0.000	0.917
3	258965.704	259861.535	259445.248	0.000	0.000	0.918
4	251102.357	252299.078	251742.969	0.000	0.000	0.897
5	248056.765	249554.376	248858.446	0.000	0.000	0.881
6	244713.479	246511.979	245676.228	**0.227**	0.000	0.891
7	243156.948	245256.339	244280.765	**0.126**	0.000	0.889
Only financial worries						
1	31153.713	31344.879	31208.359	‐	‐	‐
2	28159.881	28546.659	28270.444	0.000	0.000	0.927
3	27399.787	27982.176	27566.266	0.023	0.000	0.898
4	26855.108	27633.109	27077.504	**0.189**	0.000	0.893
5	26699.517	27673.129	26977.83	**0.618**	0.000	0.906

*Note*: Bold values highlight nonsignificant *p*‐values for Lo–Mendell–Rubin likelihood ratio test (LMR‐LRT) and bootstrapped likelihood ratio test (BLRT), which indicate support for class solutions.

Abbreviations: AIC, Akaike's information criterion; BIC, Bayesian information criterion; SABIC, sample‐size‐adjusted BIC.

#### Predictors of the latent class membership

Univariate analysis revealed significant differences in grade, gender, stress response screening results, and PSI subscale scores (*p* < 0.01–0.001; Table 2) as well as significant differences in the frequency of money worry across the five latent classes of the overall student sample (*χ* ^2^ = 102.68, *p* < 0.001). Further multinomial logistic regression analysis was conducted using predictors of class membership, with the minimal SR class as the reference class. Table 5 presents the results, showing that, compared to Grade 12, Grades 10 and 11 students were more likely to be assigned to the high SR & low SS class (odds ratio [OR] = 1.24–1.45, *p* < 0.01–0.001). Female students were more likely to be classified in the high SR & high SS class, with the odds being 2.38 times higher than for male students (OR = 2.38, *p* < 0.001), and they were less likely to be in the low SR & SS class (OR = 0.55, *p* < 0.001). Students with money worries were more likely to be classified in the high SR & SS class, and the OR was significantly higher than that for all other comparisons (OR = 3.78, *p* < 0.001).

#### Score comparison of participants with money worries and without any worries

**Table 5:** Multinomial regression of predictors by latent classes in the overall sample (*n* = 6894).

Demographic variables (Reference group: Class 5. Minimal SR)	Class 1	Class 2	Class 3	Class 4
	High SR & low SS	High SR & SS	Moderate SR	Low SR & SS
	OR (95% CI)	OR (95% CI)	OR (95% CI)	OR (95% CI)
Grade				
Grade 10, ref.: Grade 12	1.24* (1.02–1.51)	0.82 (0.66–1.01)	1.07 (0.90–1.27)	1.25* (1.00–1.56)
Grade 11, ref.: Grade 12	1.45** (1.18–1.80)	1.08 (0.86–1.34)	1.10 (0.90–1.27)	1.41** (1.12–1.79)
Gender				
Female, ref.: Male	1.57*** (1.33–1.84)	2.38*** (1.97–2.84)	1.62*** (1.40–1.87)	0.55*** (0.45–0.66)
Other, ref.: Male	2.38*** (1.60–3.54)	2.17** (1.31–3.43)	1.47 (0.97–2.21)	1.30 (0.83–2.03)
Results for screening				
Financial worries	3.78*** (2.87–4.98)	2.02*** (1.45–2.82)	1.67** (1.24–2.25)	1.74** (1.22–2.47)

* *p* < 0.05

** *p* < 0.01

*** *p* < 0.001.

Table 6 shows the statistically significant differences between participants with money worries and without any worries. Mean scores of the PSI subscales for the participants with money worries—SR (20.1 ± 10.1, mean ± standard deviation) and ST (13.6 ± 6.5)—were higher than those for participants without any worries—SR (8.7 ± 8.4 by the two‐tailed *t*‐test, *p* < 0.001, *η* ^2^ = 0.244) and ST (8.1 ± 5.9, *p* < 0.001, *η* ^2^ = 0.138)—and the effect size was large.

**Table 6:** Score comparison of participants with money worries and without any worries.

	With money worries		Without any worries		Statistics (T/*χ* ^2^)	*p*	Effect size^c^
	*n* = 630 Mean (SD)		*n* = 1816 Mean (SD)				
Scores of PSI stress reaction (SR)							
Total	20.10	(10.08)	8.65	(8.35)	28.050	<0.001^a^	0.244
Depression	6.40	(3.89)	2.52	(2.82)	26.774	<0.001^a^	0.227
Helplessness	7.83	(3.71)	3.80	(3.42)	24.886	<0.001^a^	0.202
Irritability	5.87	(4.02)	2.33	(3.07)	22.934	<0.001^a^	0.177
Scores of PSI stressor (ST)							
Total	13.63	(6.54)	8.09	(5.88)	19.779	<0.001^a^	0.138
Friends	1.45	(1.99)	0.50	(1.24)	13.956	<0.001^a^	0.074
School	1.69	(2.30)	0.65	(0.58)	12.902	<0.001^a^	0.064
Academic	5.44	(2.93)	3.48	(2.88)	14.633	<0.001^a^	0.081
Carrier	5.05	(2.43)	3.46	(2.62)	13.361	<0.001^a^	0.068
Scores of PSI social support (SS)							
Total	21.13	(8.31)	26.43	(9.17)	−12.807	<0.001^a^	0.063
Friends	7.91	(3.17)	9.27	(3.19)	−9.245	<0.001^a^	0.034
School teacher	6.03	(3.31)	7.98	(3.50)	−12.203	<0.001^a^	0.057
Family	7.19	(3.66)	9.18	(3.35)	−12.562	<0.001^a^	0.061

	*n* (%)		*n* (%)				
Grade							
Grade 10	222	(35.2)	762	(42.0)	9.0472	0.011^b^	0.061
Grade 11	168	(26.7)	449	(24.7)			
Grade 12	240	(38.1)	605	(33.3)			
Gender							
Male	292	(46.3)	981	(54.0)	11.0784	<0.01^b^	0.067
Female	309	(49.0)	760	(41.9)			
Other	29	(4.6)	75	(4.1)			
Results for screening							
Normal stress	504	(80.0)	1784	(98.2)	257.4831	<0.001^b^	0.324
High stress (A)	126	(20.0)	32	(1.8)			
High stress (B)	0	(0.0)	0	(0.0)			

^a^ *t*‐test.

^b^ Chi‐square (*χ* ^2^).

^c^ Cramer's *V* for categorical and eta‐squared for continuous variable.

The mean of the SS scores for the participants with money worries—SS (21.1 ± 8.3)—was lower than that for the participants without any worries—SS (26.4 ± 9.2, *p* < 0.001, *η* ^2^ = 0.063)—and the effect size was medium. Moreover, statistically significant differences were found between the frequencies of grade (*χ* ^2^ = 9.05, *p* = 0.01, *V* = 0.06) and gender (*χ* ^2^ = 11.08, *p* < 0.01, *V* = 0.07) and the results for stress screening (*χ* ^2^ = 257.48, *p* < 0.001, *V* = 0.32); the effect sizes were weak to medium.

### Subsample of students with money worries

#### Model selection and labeling of the identified latent class

Similarly to the model selection for the overall sample, the optimal number of latent classes for the money worries subsample was determined. AIC and SABIC values decreased as the number of latent classes increased from two to five for the money worries subsample (Table 4). The BIC value was lowest for the four‐class model; however, the LMR‐LRT indicated that a three‐class model fit the data better than a four‐class model (*p* < 0.001). Therefore, we selected a three‐class model.

**FIGURE 2:**
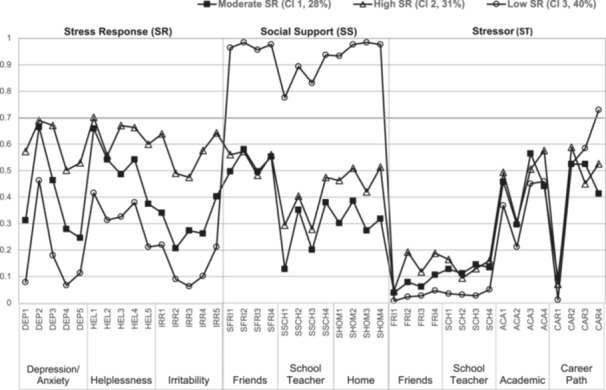
Latent classes of the subsample of students with money worries (*n* = 630). ACA, academic; CAR, career path; DEP, depression/anxiety; FRI, friends; HEL, helplessness; IRR, irritability; SCH, school teacher; SFRI, support from friends; SHOM, support at home; SSCH, support from school teacher.

For the subsample of students with money worries, class sizes ranged from 28.4% (Class 1, *n* = 179) to 40.5% (Class 3, *n* = 255) of the total sample (Table 3). In the SR domains shown in Figure 2, Class 1 exhibited moderate ratings for items, Class 2 showed relatively high ratings, and Class 3 displayed relatively low ratings. Based on these characteristics, the three classes were labeled according to their SR: Class 1 was labeled as “moderate SR,” Class 2 as “high SR,” and Class 3 as “low SR.”

#### Predictors of the latent class membership

Among the three latent classes, univariate analysis revealed significant differences in gender ($\chi^{2}$ = 13.57, *p* < 0.01), stress response screening results ($\chi^{2}$ = 295.77, *p* < 0.001), and PSI subscale scores (*F* = 12.502–538.99, *p* < 0.001), but no significant difference in grade (*p* = 0.41; Table 3). Further multinomial logistic regression analysis using predictors of class membership (Table 7) indicated that, compared to the low SR and moderate SR classes, there were no significant differences in grade. However, female students (OR = 1.76–2.05, *p* < 0.01) and students identifying as another gender (OR = 2.26–4.05, *p* < 0.05) were more likely to be assigned to the high SR class than male students.

**Table 7:** Multinomial regression of predictors by latent classes in the financial worry sample (*n* = 630).

Demographic variables	Class 2. High SR vs. Class 1. Moderate SR	Class 1. Moderate SR vs. Class 3. Low SR	Class 2. High SR vs. Class 3. Low SR
	OR (95% CI)	OR (95% CI)	OR (95% CI)
Grade			
Grade 10, ref.: Grade 12	1.25 (0.46–1.37)	1.11 (0.69–1.80)	1.40 (0.88–2.22)
Grade 11, ref.: Grade 12	1.03 (0.54–1.75)	1.41 (0.85–2.35)	1.45 (0.87–2.42)
Gender			
Female, ref.: Male	2.05** (1.27–3.30)	0.86 (0.57–1.30)	1.76** (1.16–2.66)
Other, ref.: Male	4.05* (1.15–14.32)	0.56 (0.16–1.95)	2.26 (0.92–5.56)

* *p* < 0.05

** *p* < 0.01

***p < 0.001.

#### Degree of separation for each item of stressors and social support

In Figure 2, response probabilities greater than 0.7 highlight items that were strongly endorsed by each class, reflecting the similarity among individuals within the same class. The ORs presented in Table 8 identify variables that are key to distinguishing between the classes (OR > 5 or <0.2), illustrating the degree of dissimilarity among individuals across classes. Together, these tables provide insight into items that did not significantly contribute to shaping the latent classes.

**Table 8:** Degree of class separation for the sample with financial worries (*n* = 630).

Category	Item	Sub scale #	Class 1. Moderate SR vs. Class 2. High SR	Class 1. Moderate SR vs. Class 3. Low SR	Class 2. High SR vs. Class 3. Low SR
Stress response subscale items (SR)					
Depression/Anxiety	I feel sad.	DEP 1	**0.154**	**5.338**	**34.669**
	I'm somewhat worried.	DEP 2	**0.212**	2.333	**11.013**
	I feel down.	DEP 3	**0.130**	4.113	**31.640**
	I feel like crying.	DEP 4	**0.204**	**5.173**	**25.318**
	I want to be consoled.	DEP 5	**0.175**	2.555	**14.639**
Helplessness	I lack confidence in many things.	HEL 1	**0.182**	2.721	**14.986**
	I lack perseverance.	HEL 2	0.427	2.507	**5.867**
	I am thinking about things that are not good.	HEL 3	**0.154**	1.994	**12.953**
	I cannot concentrate on anything.	HEL 4	**0.201**	1.864	**9.286**
	I can't get my story or actions straight.	HEL 5	**0.168**	2.061	**12.265**
Irritability	I tend to become angry.	IRR 1	**0.113**	1.943	**17.208**
	I am unable to control my emotions.	IRR 2	**0.153**	2.674	**17.508**
	I am displeased with things.	IRR 3	**0.230**	**5.147**	**22.417**
	I am angry.	IRR 4	**0.129**	3.188	**24.719**
	I am irritable.	IRR 5	**0.136**	2.488	**18.301**
Stressor subscale items (ST)					
Friends	Friends' violence	FRI 1	***	***	**7.282**
	Swearing	FRI 2	**0.273**	3.494	**12.813**
	Friends' orders	FRI 3	***	***	**5.806**
	Doubt from friends	FRI 4	0.401	2.592	**6.462**
School teacher	Teacher's reprimands	SCH 1	***	3.864	**7.340**
	Comparison by the teacher	SCH 2	***	4.313	**5.269**
	Imposition of the teacher's ideas	SCH 3	***	**5.706**	**7.247**
	Unkindness from the teacher	SCH 4	***	3.137	**5.112**
Academic	Not understanding the lesson	ACA 1	0.434	***	3.261
	Unable to answer when called on in class	ACA 2	***	***	2.458
	Poorer grades than friends	ACA 3	***	***	2.429
	Poor grades	ACA 4	**0.281**	***	3.554
Career path	Change of career path	CAR 1	***	**7.845**	**8.570**
	Uncertainty about the career path	CAR 2	0.334	***	3.056
	Talking about the career path with friends	CAR 3	***	***	***
	Talking about the career path with family	CAR 4	0.355	0.281	***
Social support subscale items (SS)					
Friends	Friends: Awareness and encouragement	SFRI 1	***	**0.037**	**0.098**
	Friends: Support for mistakes	SFRI 2	***	**0.019**	**0.039**
	Friends: Understanding of feelings	SFRI 3	***	**0.048**	**0.081**
	Friends: Support for worries	SFRI 4	***	**0.029**	**0.062**
School teacher	Teachers: Awareness and encouragement	SSCH 1	**0.248**	**0.041**	**0.166**
	Teachers: Support for mistakes	SSCH 2	***	**0.058**	**0.126**
	Teachers: Understanding of feelings	SSCH 3	0.432	**0.050**	**0.115**
	Teachers: Support for worries	SSCH 4	0.391	**0.040**	**0.102**
Home	Home: Awareness and encouragement	SHOM 1	0.301	**0.032**	**0.105**
	Home: Support for mistakes	SHOM 2	0.344	**0.017**	**0.050**
	Home: Understanding of feelings	SHOM 3	***	**0.008**	**0.024**
	Home: Support for worries	SHOM 4	**0.266**	**0.014**	**0.051**

*Note*: Odds ratios >5 or <0.2 are bolded to signify a high degree of class separation.^55^, ^56^

Specifically, all items related to academic stressors were the least effective in distinguishing the latent classes, as their endorsement rates across all three classes did not exceed the 0.7 thresholds and did not significantly contribute to class separation. In contrast, items in the SR and SS subscales, and stressors related to friends, school teachers, and career path changes were strongly endorsed across all latent classes and significantly contributed to the distinction between classes.

In Table 8, compared to students in the low SR class, those in the high SR class were more likely to have depression/anxiety (OR = 11.013–34.669) and stressors related to friends and school teachers (OR = 5.112–12.813) and were less likely to perceive social support from family members and friends (OR = 0.024–0.098).

Students in the moderate SR class were more likely to report stressors such as “Imposition of the teacher's ideas” (OR = 5.706) and “Change of career path” (OR = 7.845), and were less likely to perceive social support from all sources (OR = 0.008–0.058) compared to the low SR class. These ORs represented the lowest values among all comparisons.

In the comparison between the high SR class and the moderate‐SR classes, 10 items from the SR subscale, especially in the domain of irritability subscales (OR = 0.113–0.153), showed a significant degree of separation; however, no items from the ST or SS subscales did. However, “Swearing” (OR = 0.273) and “Poor grades” (OR = 0.281) from the ST subscale, and “Teachers: Awareness and encouragement” (OR = 0.248) and “Home: Support for worries” (OR = 0.266) from the SS subscale were close to the significance threshold.

## DISCUSSION

To the best of our knowledge, this is the first study to combine a school‐based stress check system with LCA to identify students at risk for mental health and economic challenges, and to analyze their characteristics based on stress responses, social support, and school‐related stressors. Our findings indicate that students with money worries were more likely to experience high‐stress responses, high stressors, and low social support than students without any worries. Additionally, distinct differences were observed in stress responses, stressors, and social support among the three latent classes in the sample of students with money worries. These results suggest that web‐based stress checks in high schools can play a key role in understanding students' backgrounds, serving as an intervention not only to enhance mental health but also to prevent academic underachievement and emotional problems by screening for money worries. Money worries often lead to self‐stigma and shame, making it difficult to seek help, and the invisibility when providing support is a big barrier to their future life.^57^ To reduce the invisibility, we conducted two latent class analyses for the entire student population and, specifically, for the group experiencing money worries. This study attempted to delineate the psychosocial characteristics of students experiencing money worries in actual high school life from those of other students.

### Screening students in poverty

In this study's overall sample, 9.1% of students were identified as having money worries. Of these, 20% were in the high‐stress group, while 12.7% of the overall sample were classified in this group. Students with money worries were 3.78 times more likely to be in the high‐stress group than in the minimal stress group. Although the psychological scales and survey items used in previous studies differ, preventing a direct comparison with our results, this study's findings suggest that money stress contributes to poor mental health. Therefore, the present study's hypothesis—that money worries are associated with high‐stress levels in adolescents—was supported.

The relative child‐poverty rate in Japan at the time was 11.5%.^2^ The study results may include children living in relative poverty; however, we were only able to address subjective money worries (i.e., perceived financial stress) and not those based on the family's actual income. Because we prioritized feasibility in schools, we avoided setting multiple questions regarding specific economic‐stress situations, such as household financial difficulties or lack of pocket money.

Therefore, given the limitations described later, the findings should be viewed with caution. Due to the less‐studied area of adolescent mental health and pocket money,^58^, ^59^, ^60^ we discuss our results relative to those of studies on perceived financial stress and the family's low income.

### Latent class characteristics of students with money worries

The LCA results using stress response, stressors, and social support in a sample of students with money worries revealed three stress response classes: “low SR” (40.5%), “moderate SR” (28.4%), and “high SR” (31.1%). The low SR group showed high perceived social support, while the moderate and high SR groups reported low perceived social support from their friends, school teachers, and family. Based on the “stress‐buffering model of social support” by Cohen and Wills,^61^ support mitigates the harmful effects of stress for those who receive sufficient support. This model was supported in our study.

We also examined the effects of grade and gender in the subsample of students with money worries. While no significant differences were found by grade in the comparison of each class, gender differences were evident. Female students and those who identified as another gender were significantly more likely to be assigned to classes with high‐stress responses than male students. A prospective longitudinal study conducted in Sweden found that among adolescents from low‐income households, females were more likely than males to develop mental disorders at ages 16 years and 17 years. This study's findings appear to be consistent with those results.^62^ Additionally, previous research has reported that adolescents who identify as transgender or gender diverse are more likely to experience emotional distress and bullying. It is possible that similar experiences contributed to the findings in this study.^63^

### Stress responses, emotional, and behavioral problems

Previous studies have reported that individuals with financial difficulties are more likely to experience emotional and behavioral problems.^64^ The subscales in this study—depression and anxiety, helplessness, and anger—are also known to be exacerbated by poverty and financial difficulties.^65^, ^66^, ^67^ When comparing the high‐stress response group with the low‐stress response group, all items on the Stress Response subscale were significantly higher, with depression and anxiety items being particularly elevated. In the moderate stress response group, the depression and anxiety subscale item “I'm somewhat worried” and the helplessness subscale item “I lack perseverance” had response rates similar to those of the high‐stress response group. Regarding depression and anxiety, previous research indicates that individuals with the lowest income levels are 1.5 to 3 times more likely to develop depression or anxiety disorders than those with higher incomes.^68^ Similarly, low perseverance has been linked to reduced academic resilience and lower academic performance, with financial hardship increasing the likelihood of high school dropout.^69^, ^70^

### Stressors and social support related to school life

In this study, the low SR group not only reported fewer stressors related to friendships, school, and career choices but also perceived higher levels of SS. Conversely, the moderate and high SR groups experienced greater stressors in these domains while reporting lower levels of SS. Relationships can serve as both a stress source and an SS source.^71^ During adolescence, poverty and financial difficulties can heighten the risk of social isolation and bullying.^72^ However, previous studies suggest that students who experience warm parental relationships and maintain strong connections with friends and teachers exhibit fewer psychological and behavioral problems and achieve better academic performance.^73^, ^74^, ^75^ Consistent with these findings, this study also observed a correlation between higher stress responses and increased stressors related to friendships and school, as well as an inverse correlation between stress responses and social support.

### Academic and career‐related stressors

This study found no significant differences in academic‐related stress among the three latent classes. Previous studies have reported that academic stress is a risk factor for depression, anxiety symptoms, and school dropout during adolescence^76^, ^77^, ^78^ and is often significantly associated with low family economic status. However, in this study, no significant association was observed between academic stress and class grouping based on stress levels. This finding may suggest the presence of a common factor in all latent classes. Future research should include comparisons with a control group using a controlled sample size to investigate this relationship further. Additionally, among the career‐related stressors, only the item “I was told to change my career path” showed a significant difference in the degree of separation between the classes. In previous research, this has been examined under the concept of “self‐efficacy in career decision‐making” among high school students, although its connection to a low‐income family background remains under discussion.^79^ A study conducted in Hong Kong found that career education interventions for adolescents and parents from low‐income families were effective.^80^ Along with poor academic performance and chronic absenteeism, other risk factors for high school dropout include coming from a low‐income or single‐parent household, receiving little support from parents or teachers, and experiencing negative peer influences.^81^ Given these factors, the simultaneous assessment of mental health, social support, and school‐related stress—such as through the PSI scale used in this study—may serve as an important approach for preventing school dropout.

### Support strategies based on latent class analysis

Currently, in Japan, when students facing money worries are identified at school, the main support provided focuses on monetary assistance and livelihood support. Financial support includes assistance from administrative bodies such as local governments for tuition, commuting expenses, school supplies, and school trip costs, as well as scholarship programs. Livelihood support includes support for free learning, children's cafeteria, and leisure activities provided by local private organizations (e.g., nonprofit organizations). When schools identify students who have money worries and high stress levels, as determined by the class classification in this study, psychological support is necessary in addition to financial and livelihood assistance. This requires collaboration with mental health professionals, including school counselors and medical doctors. In such cases, consideration must be given to providing financial support for the costs associated with visiting mental health professionals or medical institutions, as well as transportation expenses. Furthermore, if students tend to have low social support at school, educational and school counseling should assist them in resolving issues stemming from money worries, such as social isolation or friendship problems. If students tend to have low social support at home, it is necessary to collaborate with school social workers to assess the home environment and provide support involving the family. In any of these classes, when support for poverty becomes necessary, students and parents may be reluctant to disclose their financial difficulties to others.^82^ Therefore, when providing support for poverty, it is crucial to engage in ways that minimize psychological burdens such as shame, social isolation, and anxiety.

### Limitations and future research

The primary limitation of this study was the difficulty in adjusting the sample size. Because the data were primarily collected for routine mental health checkups, achieving a balanced sample in terms of gender and grade distribution was challenging from the outset. In particular, increasing the sample size of students with money worries might have allowed for the identification of additional latent classes that could otherwise be overlooked beyond the three classes identified in this study.^83^ Furthermore, our sample was collected from only public schools (65 schools) in a single prefecture, not including private schools. Future research should aim to secure a larger sample size to enable the simultaneous analysis of multiple populations and the application of stratified random sampling for improved representativeness and generalizability.^84^ Another limitation of this study is the phrasing used in the screening question for money worries. The phrase “Livelihood or money” does not explicitly reference poverty or financial hardship within the household. This indirect wording might possibly have captured high school students who were concerned about having limited pocket money. However, in the broader context of children and financial well‐being, recent research has expanded beyond poverty to examine perceived economic stress, subjective social status,^85^ material deprivation,^86^ and pocket money. These factors have been shown to influence depression, anxiety, and SS. Based on the Family Economic Stress Model, Mistry et al. (2009) conducted a survey to use three questions to assess adolescent perceptions of family economic stress, for example, “In the past 3 months, how much of a problem did your family have because your parents did not have enough money to buy things your family needs or wants?” and two questions of adolescent perceptions of financial constraints to think about the past 3 months and identify how often they had enough money “for things like clothes, school activities, or things you need” and “for doing things you and your friends like to do, such as going to movies, eating pizza, etc.” Future research should explore and validate the most effective screening phase for adolescents by testing the questions to assess the adolescents' perception of family economic stress and financial constraints.^87^

## CONCLUSION

A web‐based child stress check is a crucial tool for promoting educational equity by facilitating the screening and assessment of students' mental health risks and money worries. When combined with latent class analysis, this approach provides multidimensional insights into individual students, enabling more targeted support in high school settings.

## AUTHOR CONTRIBUTIONS

Yurika Namihira, Miyuki Furukawa, Ayako Tsuchiya, and Eiji Shimizu collected the data. Yurika Namihira, Miyuki Furukawa, and Yoshikazu Noda conducted statistical analyses and interpretation of data. Yoshikazu Noda, Hironori Shimada, and Eiji Shimizu critically revised the manuscript. Seiichiro Hori, Takako Koshiba, and Eiji Shimizu supervised the study. All authors contributed to the article and approved the submitted version.

## CONFLICT OF INTEREST STATEMENT

The authors declare no conflicts of interest.

## ETHICS APPROVAL STATEMENT

The studies involving human participants were reviewed and approved by the Ethics Committee of Chiba University (ID: M10631). The patients/participants provided their informed consent to participate in this study.

## CLINICAL TRIAL REGISTRATION

N/A.

## PATIENT CONSENT STATEMENT

Informed consent was obtained from parents before the study commenced. Children participated using their own digital devices after reviewing information about the study and providing informed consent.

## Data Availability

The data that support the findings of this study are available on request from the corresponding author. The data are not publicly available due to privacy or ethical restrictions.
